# Supplementary material for: Stacking Model‐Based Classifiers for Dealing With Multiple Sets of Noisy Labels
Source: Biom J. 2025 Mar 12;67(2):e70042. doi: 10.1002/bimj.70042 (PMC11898607; doi:10.1002/bimj.70042)
Supplement: Supplementary file 1 — Supporting Information [file BIMJ-67-e70042-s001.zip › density-based-ensemble-model-main/competitors_real_data/netlab-master/netlab-master/help/glmgrad.htm]

Netlab Reference Manual glmgrad


# glmgrad

## Purpose

Evaluate gradient of error function for generalized linear model.

## Synopsis

```
g = glmgrad(net, x, t)
[g, gdata, gprior] = glmgrad(net, x, t)
```

## Description

`g = glmgrad(net, x, t)` takes a generalized linear model
data structure `net`
together with a matrix `x` of input vectors and a matrix `t`
of target vectors, and evaluates the gradient `g` of the error
function with respect to the network weights. The error function
corresponds to the choice of output unit activation function. Each row
of `x` corresponds to one input vector and each row of `t`
corresponds to one target vector.

`[g, gdata, gprior] = glmgrad(net, x, t)` also returns separately
the data and prior contributions to the gradient.

## See Also

`glm`, `glmpak`, `glmunpak`, `glmfwd`, `glmerr`, `glmtrain`

---

**Pages:**
Index


---

Copyright (c) Ian T Nabney (1996-9)
